# Energy drink consumption, sleep behavior, and food choices of Icelandic adolescents

**DOI:** 10.29219/fnr.v70.12190

**Published:** 2026-01-29

**Authors:** Runa Stefansdottir, Arna O. Gunnarsdottir, Bjorn J. Hjalmarsson, Ingibjorg Gunnarsdottir, Erlingur Johannsson

**Affiliations:** 1 Center of Sport and Health Sciences, Department of Health Promotion, Sport and Leisure Studies, School of Education, University of Iceland, Reykjavík, Iceland; 2 Faculty of Food Science and Nutrition, School of Health Sciences, University of Iceland, Reykjavik, Iceland; 3 Unit for Nutrition Research, Landspitali University Hospital, Reykjavík, Iceland; 4 Department of Sport, Food and Natural Sciences, Western Norway University of Applied Sciences, Bergen, Norway

**Keywords:** *energy drinks*, *youth*, *sleep duration*, *food choices*, *caffeine*

## Abstract

**Background:**

The consumption of energy drinks has increased in the last decades, especially among adolescents. Caffeine and its effects on sleep are well known, but less is known about the timing of the consumption and its association with sleep and food choices.

**Objective:**

The objective of this study was to evaluate the energy drink consumption, sleeping behavior, and food choices in Icelandic adolescents.

**Design:**

A total of 171 participants (64 boys, 107 girls, aged 17–18 years) completed an online questionnaire on sleep, food choices, and energy drink consumption. Independent sample *T*-tests, Mann-Whitney *U* tests, and Chi-square tests were used to assess group differences.

**Results:**

Overall, 57% reported drinking energy drinks, with higher rates among girls than boys (63 vs. 48%). Energy drink consumers were more likely to report sleeping 6 h or less. This was especially true for those drinking energy drinks after 3 PM, compared to those who avoided them after 3 PM. Participants who consumed energy drinks also ate fewer nutritious foods (fruits, vegetables, dairy, fish) and consumed more soft drinks, coffee, and alcohol compared to non-energy drink consumers.

**Discussion:**

The results show that energy drink consumption is frequent among Icelandic 17-year-olds, particularly among girls. Consumers were more likely to report shorter sleep durations, especially when drinking after 3 PM, and had poorer dietary habits, including lower intake of nutritious foods and higher consumption of soft drinks, coffee, and alcohol.

**Conclusion:**

Future research should explore the long-term effects of these behaviors and assess interventions to reduce energy drink use and promote healthier habits in adolescents.

## Popular scientific summary

- Energy drink consumption was common among Icelandic adolescents, with more than half reporting regular use.
- Adolescents who consumed energy drinks, particularly later in the day, were more likely to report shorter sleep duration on school days.
- Energy drink consumers also reported poorer dietary habits, including lower intake of nutritious foods and higher consumption of soft drinks, coffee, and alcohol.

The popularity of energy drinks has risen significantly over the past few years, particularly among adolescents and young adults (1). Energy drinks are non-alcoholic beverages that typically contain a large amount of caffeine. In addition to caffeine, they also include sugar, vitamins, minerals, and amino acids, although their content varies between products (1, 2). Adolescents’ exposure to caffeine has been raising concerns due to the fact that caffeine is a psychoactive stimulant that has been linked to alterations in cognitive, physiological, psychological, and cardiovascular functions in adolescents (3, 4). Furthermore, high caffeine intake has been associated with poorer sleep health, including later bedtimes, shorter time in bed (5) and daytime sleepiness (6). The European Food Safety Authority (EFSA) states that adolescents who consume caffeine at a dose of 1.4 mg per kilogram of body weight are more likely to experience longer sleep latency and shorter sleep duration, especially when consumed close to bedtime (2).

Sleep is essential for overall health and well-being (7), as well as cognitive, physical, and emotional health among adolescents and young adults (8). Therefore, the National Sleep Foundation (NSF) recommends at least 8–10 h of sleep for healthy teenagers, ages 14–17 and emphasizes regularity and structure in sleep schedules (9). Inadequate sleep has been linked to a numerous negative effect on physical health (10), mental well-being (11), cognitive abilities (12), and a recent study reported that both later bedtime and higher night-tonight variability in total sleep time were negatively associated with academic performance (13). Conversely, extending adolescents sleep duration has demonstrated the potential to enhance metabolic factors and dietary habits (14), and improve cognitive function (15).

Previous studies have reported that energy drink consumption is associated with poorer dietary quality in general (16) and increased intake of consumption of high-sugar and fried foods (3). Although the effect of caffeine intake on sleep and its association with consumption of unhealthy diet is quite well known, less is known about the timing of energy drink consumption in adolescents as well as the association between the timing of consumption and sleep duration.

The main objective of the study was to evaluate the energy drink consumption, sleeping behavior, and food choices in Icelandic adolescents. Specific aims included: 1) to compare different sleep durations among those who consume and those who do not consume energy drinks, 2) to explore sleep duration with amount of caffeine and the time-of-day energy drink consumed and 3) to compare food choices among those who consume and those who do not consume energy drinks.

## Methods

### Sample and data collection

Data were collected between March and May 2021, in three secondary schools, all located in the capital area of Reykjavik, Iceland. The data collection was organized in consultation with school administrators in each school individually and each student. A total of 420 students born in 2003 (aged 17–18 at the time of data collection) were invited to participate from the three secondary schools. A total of 184 students agreed to participate (participate rate 44%) in the study. Out of these, 171 students (107 girls) finished answering the self-reported questionnaires about sleep and food choices, along with detailed questions about the frequency and timing of energy drink consumption. Written informed consent was obtained from students of consenting age and the parents consented if students were younger than 18 years old. The study was approved by the Icelandic Data Protection Authority and the National Bioethics Committee (VSN b2021010039/03.01) and was carried out with the permission of the school’s principals.

### Measures

The questionnaire was based on a previously validated questionnaire used by The Directorate of Health (17) and distributed via Qualtrics XM survey software. Participants were asked about their gender, weight (with the following questions: ‘What is your height when you stand only wearing socks (in centimeters)?’) and height (with the question ‘What is your body weight without clothes (in kilograms)?’). Information on weight and height was used to calculate body mass index (BMI) and was then calculated by dividing weight by height squared (kg/m²).

*Sleep behavior.* Self-reported total sleep duration was measured with the question: 1) How many hours of sleep do you usually get per night a) during weekdays and b) on weekends, with seven answer option: ‘4 hours or less’, ‘5 hours’, ‘6 hours’, ‘7 hours’, ‘8 hours’, ‘9 hours’ or ‘10 hours or more’. Participants were asked to round up their sleep time to the nearest hour and report on most days during the last month. Bedtime on weekdays and weekends was calculated from the question: a) ‘What time do you usually go to bed on weekdays’ and b) ‘What time do you usually go to bed on weekends’, with the answer options: ‘20:00’, ‘21:00’, ‘22:00’, ‘23:00’, ‘00:00’, ‘01:00’, ‘02:00 or later’. Similarly, risetime on weekdays and weekends was calculated from the question: a) ‘What time do you usually wake up on weekdays’ and b) ‘What time do you usually wake up on weekends’, with the answer options: ‘05:00’, ‘06:00’, ‘07:00’, ‘08:00’, ‘09:00’, ‘10:00’, ‘11:00 or later’. This study highlighted participants with sleep duration of ≤6 h as a conservative marker of chronic short sleep. While the NSF recommends 8–10 h of sleep for adolescents aged 14–17, and considers <7 h insufficient, it also notes that 7–11 h may be appropriate depending on the individual (9). Prior studies have emphasized that sleep durations of ≤6 h are particularly associated with adverse cognitive, psychological, and physical health outcomes in adolescents (7, 12, 15).

*Energy drink consumption.* Information about general intake of energy drinks was gathered by a ‘yes/no’ questions about energy drink consumption. Those who responded ‘yes’ received further questions providing detailed information about the type, amount and timing of energy drink consumption. The 18 most popular energy drinks on the Icelandic marked were listed along with an open-ended answer for other energy drinks. For each type the participants were asked to estimate the frequency of consumption, with the answer options: ‘never’, ‘1–2 times in a month’, ‘3–4 times in a month’, ‘1–2 times per week’, ‘3–4 times per week’, ‘5–6 times per week’, ‘1–2 times a day’ and ‘3 times or more a day’. This question was followed by questions regarding the timing of energy drink consumption. The weekdays were divided into morning (06–12 AM), early afternoon (12–3 PM), late afternoon (3–6 PM), early evening (6–9 PM), late evening (21–00 PM) and night (00–06 AM), with the answer options: ‘never’, ‘1–2 times per week’, ‘3–4 times per week’, ‘5 times or more a week’. To calculate the total caffeine consumption from drinks, we used portion sizes and caffeine content for the most popular energy drinks available in the Icelandic market. These are listed in Supplement Table 1 along with the number (and %) of participants reporting consuming each type. Total caffeine intake was compared with safety limits suggested by EFSA (2).

*Food consumption.* Participants were also asked to report their average consumption of food and drinks in the past 3 months. The questionnaire covered selected food groups: Fruit and berries, vegetables, milk and dairy products, vegetable dishes, fish, meat, processed meat products, chips and potatoes, cookies and cake, chocolate and candy, sugar-free soda, soda with sugar, dark cola drinks, sports drinks, coffee, and alcohol. The answer options for each category were ‘never’, ‘1–2 times in a month’, ‘3–4 times in a month’, ‘1–2 times per week’, ‘3–4 times per week’, ‘5–6 times per week’, ‘1–2 times a day’, ‘3–4 times a day’, and ‘five times or more in a day’.

### Statistical analysis

Data analysis was conducted in SPSS (SPSS Statistics version 28) and JAMOVI 2.3.21 (The Jamovi Project). Data were reported as mean (standard deviation) for normally distributed variables and as the median and percentiles for skewed variables (frequency of dietary intake). The normality of continuous variables was assessed using the Kolmogorov–Smirnov test. Dichotomous variables are reported as frequencies and percentages (%). For continuous variables, an independent sample *T*-test was used to test the significance of differences between the two groups of normally distributed variables, and the Mann–Whitney U test was used to assess the differences among skewed variables, applying a post hoc Bonferroni correction for multiple comparisons. The Chi-square test was used to test differences in dichotomous variables across groups. *P* < 0.05 was considered statistically significant. All reported *P*-values are two-sided.

## Results

Demographic characteristics of the study sample are presented in Table 1. The study sample included 107 girls and 64 boys who had valid data. In total, 57% reported drinking energy drinks, while higher frequency was seen among girls compared with boys (63 vs. 48%, *P*-value <0.001). Total sleep duration on school days was 6.5 (1.1) h/night and 8.3 (1.1) h/night on weekends. Overall, no gender difference was found in any of the sleep parameters (Table 1).

**Table 1:** Study sample descriptives and comparison between boys and girls

Variables	All (*n* = 171)	Boys (*n* = 64)	Girls (*n* = 107)	*P*-value
	Mean (SD)	Mean (SD)	Mean (SD)	
**Height (cm)**	174 (8.9)	182 (6.2)	168 (5.9)	**<0.001**
**Weight (kg)**	67.6 (11.5)	73.7 (10.4)	64 (10.6)	**<0.001**
**BMI (kg/m** ^2^ **)**	22.4 (3.4)	22.2 (3.1)	22.5 (3.5)	0.598
**Energy drinks consumption (%)**	57.3%	48.4%	62.6%	**<0.001**
**Total sleep duration weekdays (hours)**	6.5 (1.1)	6.5 (1)	6.5 (1.2)	0.824
**Bedtime weekdays (hh:mm, SD in hours)**	23:48 (1.1)	23:54 (1.2)	23:48 (1.1)	0.445
**Risetime weekdays (hh:mm, SD in hours)**	07:28 (0.9)	07:25 (0.8)	07:29 (0.9)	0.634
**Total sleep duration weekend (hours)**	8.3 (1.1)	8.4 (1)	8.2 (1.2)	0.061
**Bedtime weekend (hh:mm, SD in hours)**	01:12 (1)	01:18 (0.9)	01:06 (1)	0.453
**Risetime weekend (hh:mm, SD in hours)**	10:06 (1)	10:06 (1)	10:06 (1)	0.921

*n*, number of participants; BMI, body mass index. Mean (SD); time shown in hh:mm, SD in hours.

The *P*-values are the result of *T* tests or Mann-Whitney *U* test depending on variable distribution (applying post hoc Bonferroni for multiple comparisons), comparing boys and girls. Boldface type indicates significant difference.

The responses from participants about how frequently they consume energy drinks at different times during school days are summarized in Table 2. Energy drink consumption in the early evening (6–9PM) was reported by 25% of the boys and 45% of the girls. Energy drink consumption later in the evening or at night was less common, but still 27 and 12% of the girls and boys reported late evening or night consumption, respectively.

**Table 2:** Number of participants reporting energy drink consumption at different times during the day on schooldays*

Time	Never, *n*	1–2 times per week, *n*	3–4 times per week, *n*	5 times per week, *n*
Boys (*n* = 31)				
Morning (06–12am)	11	7	9	4
After lunch (12–3PM)	7	13	9	2
Afternoon (3–6PM)	15	10	5	1
Early evening (6–9PM)	23	4	4	0
Late evening (9PM–12AM)	27	3	1	0
Night (12–06AM)	29	2	0	0
Girls (*n* = 67)				
Morning (06–12am)	27	19	15	6
After lunch (12–3PM)	24	27	13	3
Afternoon (3–6PM)	34	26	5	2
Early evening (6–9PM)	37	23	6	1
Late evening (9PM–12AM)	49	16	1	1
Night (12–06AM)	59	7	0	1

* Only showing participants reporting energy drink consumption (*n* = 98).

Table 3 shows the estimated mean caffeine intake from different sources, total caffeine intake from drinks and the estimated daily caffeine intake per kg body weight. Among boys who did not report consuming energy drinks, the highest caffeine intake per body weight was 0.4 mg/kg/day, while 55 and 32% of boys who reported drinking energy drinks exceeded the EFSA limits (2) of 1.4 and 3 mg caffeine/kg/day, respectively. Both boys and girls who reported drinking energy drinks had higher caffeine intake from other sources compared to those who do not consume energy drinks (Table 3). For girls reporting energy drink consumption, 46% had greater total caffeine intake from drinks with more than 1.4 mg caffeine/kg/day and 18% higher than 3 mg/kg/day. One heavy coffee drinker was observed in the group not reporting energy drink consumption, with a caffeine intake of 2.4 mg/kg/day, but otherwise none had an intake higher than 0.7 mg/kg/day.

**Table 3:** Caffeine quantity from different sources divided between gender and those who reported drinking energy drinks and those who do not

Caffeine from different sources, mg/day			Dark coloured soft drinks	Coffee	Energy drinks with caffeine content ≤105 mg in one portion	Energy drinks with caffeine content > 105 mg in one portion	Total caffeine from drinks	Total caffeine mg/kg/day
**Boys drinking ED (*n* = 31)**	Mean		14	18	55	58	144	2.07
	Std. deviation		18	40	44	80	109	1.65
	Percentiles	25	2	0	18	9	48	0.80
		50	8	0	40	20	114	1.45
		75	19	13	85	81	224	3.27
		95	56	150	165	283	399	6.07
**Boys not drinking ED (*n* = 33)**	Mean		3	1	0	0	3	0.05
	Std. deviation		7	2	0	0	7	0.10
	Percentiles	25	0	0	0	0	0	0.00
		50	0	0	0	0	0	0.00
		75	2	0	0	0	3	0.05
		95	29	8	0	0	29	0.38
**Girls drinking ED (*n* = 67)**	Mean		8	17	69	21	115	1.77
	Std. deviation		18	39	71	50	100	1.59
	Percentiles	25	0	0	17	0	48	0.68
		50	2	0	48	0	88	1.34
		75	8	5	88	20	170	2.58
		95	29	150	202	128	316	4.28
**Girls not drinking ED (*n* = 40)**	Mean		2	6	0	0	8	0.14
	Std. deviation		6	24	0	0	25	0.41
	Percentiles	25	0	0	0	0	0	0.00
		50	0	0	0	0	0	0.00
		75	2	0	0	0	7	0.09
		95	18	21	0	0	29	0.72

Boys consuming more than 3 mg/kg/day reported shorter total sleep duration on weekdays compared with those consuming less than 3 mg/kg/day, but no difference was seen during weekends nor with lower caffeine intake (Table 4). Among girls, a difference in sleep duration was observed at an intake level of 1.4 mg/kg/day. Girls with an intake of ≥3.0 mg/kg/day appeared to report longer sleep durations on weekends compared to those with lower intakes; however, this subgroup was small, and results should be interpreted with caution.

**Table 4:** Mean (SD) hours of sleep on weekdays and weekends by caffeine intake (mg/kg/day)

Sleep variables		<1.4 mg/kg/day (*n* = 47)	≥1.4 mg/kg/day (*n* = 17)	P-value	<3 mg/kg/day (*n* = 54)	≥ 3 mg/kg/day (*n* = 10)	*P*-value
**Boys**							
	Hours of sleep weekday, mean (SD)	7.0 (0.9)	6.5 (1.1)	0.129	7.0 (1.0)	6.1 (1.0)	**0.007**
	Hours of sleep weekend, mean (SD)	8.6 (1.0)	8.8 (1.0)	0.495	8.7 (1.0)	8.4 (1.2)	0.935
		**<1.4 mg/kg/day (*n* = 75)**	**≥1.4 mg/kg/day (*n* = 32)**	***P*-value**	**<3 mg/kg/day (*n* = 95)**	**≥ 3 mg/kg/day (*n* = 12)**	
**Girls**							
	Hours of sleep weekday, mean (SD)	7.0 (1.2)	6.4 (1.0)	**0.003**	6.9 (1.2)	6.2 (1.1)	**0.029**
	Hours of sleep weekend, mean (SD)	8.2 (1.1)	8.6 (1.0)	0.09	8.2 (1.2)	9.0 (0.7)	**0.022**

Caffeine doses of around 1.4 mg/kg of body weight may delay sleep onset and shorten sleep duration in some children and adolescents, especially when consumed near bedtime. A daily intake of up to 3 mg/kg is proposed as a safe level for regular caffeine consumption in this age group.^2^

**Fig. 1:**
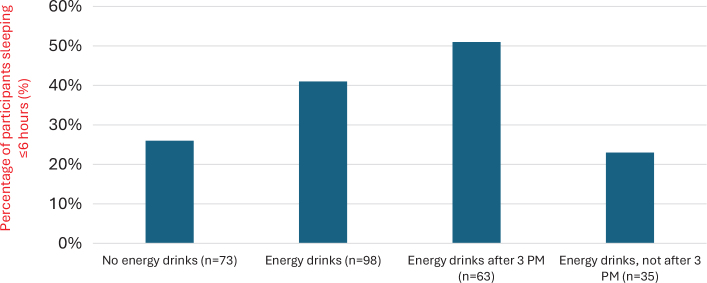
Percentage of participants sleeping ≤6 h on weekdays by energy drink consumption status and timing. Y-axis shows the proportion of participants (%). Sample sizes for each group are noted: no energy drinks (*n* = 73), energy drinks (*n* = 98), after 3 PM (*n* = 63), not after 3 PM (*n* = 35).

Figure 1 shows the proportion of participants sleeping 6 h or less on weekdays, according to energy drink consumption and the timing of consumption. In general, those reporting consuming energy drinks were likelier to report total sleep duration of 6 h or less (*P* = 0.044). Furthermore, the percentage of participants with 6 h or less sleep duration was higher in the group drinking energy drinks after 3 PM compared with those reporting consuming energy drinks but never after 3 PM (*P* = 0.007). Interestingly, the percentage of participants who had a total sleep duration of 6 h or less was similar for those reporting consuming energy drinks but not after 3 in the afternoon as in the group who never drink energy drinks.

Weekly frequency of consumption of food from selected food groups was also compared between energy drink consumers and those who did not report drinking energy drinks (Table 5). The total frequency of consuming food groups that are in general considered a part of a nutritious diet (fruits, vegetables, dairy products, and fish) was significantly lower among those reporting consuming energy drinks (*P* = 0.015). Boys who reported drinking energy drinks had less frequent intake of dairy products compared with those not drinking energy drinks (5.5 vs. 10.5 times per week, *P* = 0.003) but higher frequency of soft drink coffee and alcohol consumption. Girls exhibited a similar pattern, with those consuming energy drinks reporting higher intake of soft drinks, sports drinks and alcohol.

**Table 5:** Weekly frequency of consumption of food from selected food groups

Food consumption	Boys ED (*n* = 31)			Boys not ED (*n* = 33)				Girls ED (*n* = 67)			Girls not ED (*n* = 40)			
	Median	Percentiles		Median	Percentiles			Median	Percentiles		Median	Percentiles		
		25	75		25	75	*P*-value		25	75		25	75	*P*-value
**Fruits and berries**	3.5	1.5	10.5	5.5	3.5	24.5	0.080	3.5	1.5	10.5	5.5	1.5	10.5	0.88
**Vegetables**	5.5	3.5	10.5	10.5	3.5	17.5	0.055	5.5	3.5	10.5	5.5	1.5	10.5	0.652
**Dairy products**	5.5	1.5	10.5	10.5	5.5	24.5	**0.003**	5.5	0.9	10.5	5.5	1.5	10.5	0.55
**Fish**	1.5	0.4	1.5	1.5	0.9	1.5	0.674	0.9	0.4	1.5	1.2	0.4	1.5	0.475
**Meat**	3.5	1.5	5.5	3.5	1.5	3.5	0.841	1.5	0.9	3.5	1.5	0.5	3.5	0.787
**Processed meat**	0.9	0.4	0.9	0.9	0.4	0.9	0.893	0.4	0.0	0.9	0.4	0.0	0.9	0.275
**French fries and potato chips**	1.5	0.9	1.5	0.9	0.9	1.5	0.100	0.9	0.4	1.5	0.9	0.4	1.5	0.267
**Sweets**	1.5	0.9	3.5	1.5	0.9	3.5	0.929	3.5	1.5	5.5	2.5	1.5	3.5	0.536
**Bakery goods**	0.9	0.4	1.5	1.5	0.9	2.5	0.339	1.5	0.9	1.5	1.2	0.9	1.5	0.415
**Artificially sweetened soft drinks**	0.9	0.0	3.5	0.0	0.0	0.9	**0.010**	0.9	0.0	1.5	0.0	0.0	0.4	**<0.001**
**Sugar sweetened soft drinks**	1.5	0.0	3.5	0.4	0.0	1.2	0.078	0.9	0.0	1.5	0.4	0.0	0.9	**0.009**
Dark colored soft drinks*	1.5	0.4	3.5	0.0	0.0	0.4	**<0.001**	0.4	0.0	1.5	0.0	0.0	0.4	**0.001**
**Sport drinks**	0.9	0.0	1.5	0.4	0.0	1.5	0.427	0.4	0.0	1.5	0.0	0.0	0.8	**0.044**
**Coffee**	0.0	0.0	0.9	0.0	0.0	0.0	**0.002**	0.0	0.0	0.4	0.0	0.0	0.0	0.141
**Alcoholic drinks**	0.4	0.0	0.9	0.0	0.0	0.0	**0.001**	0.4	0.0	0.9	0.0	0.0	0.4	**0.002**

The *P*-values are the result of Mann-Whitney *U* test applying post hoc Bonferroni for multiple comparisons, comparing boys who drink energy drinks vs boys who do not drink energy drinks and girls who drink energy drinks vs girls who do not drink energy drink.

* Coca Cola, Pepsi.

## Discussion

The results of this study are in line with previous studies showing that intake of caffeine is associated with total sleep duration (18, 19), but at the same time adding important information about the timing of energy drink consumption. Finally, our study adds to the current literature showing overall lower diet quality among energy drink consumers compared with non-consumers.

### Energy drink consumption, timing and quantity

This study reveals that energy drink consumption is high among Icelandic adolescents, with 57% of participants consuming energy drinks, and a higher proportion of girls consuming them compared to boys. Our results are in line with previous Icelandic data from 2020 where 48% of adolescents reported drinking at least one energy drink per day (20). These results from Iceland are alarmingly higher than recent data from other countries, where Norwegian adolescents aged 15–16 years old reported that 8.3% of the participants consumed energy drink once a week (21), 6.3% of Swedish adolescents consumed at least once a week (22), and 7.6% of US adolescents (mean age 15.4 years) consumed energy drinks on a weekly basis (23). One possible explanation for the high frequency of energy drink consumption in Iceland is their wide availability and appeal to adolescents, which may be influenced by marketing practices targeting this age group (3, 19). Furthermore, Icelandic adolescents have access to an extensive variety of energy drinks (as seen in Supplement Table 1).

The timing of energy drink consumption was interesting as we saw that the majority of participants consumed drinks after lunch or in the afternoon (12–6PM). However, 25% of the boys and 45% of the girls reported energy drink consumption in the early evening (6–9PM) and 27% girls and 12% boys reported late evening or night consumption (9PM–6AM). Recent self-report study among Icelandic students (mean age 24.1 years old) reported the majority of participants consuming their caffeinated beverages before 5 pm, and only 12% consumed after 5 pm (24). Therefore, our results indicate that younger students were more likely to consume energy drinks later in the day. Recommendations suggest that it is better to consume caffeinated beverages earlier in the day, ideally not after approximately 3 p.m., since the half-life of caffeine in healthy, non-smokers is around 4–6 h (18). As an adenosine antagonist, caffeine increases adenosine’s inhibitory action, reduces neurotransmission that affects sleep homeostasis and promotes wakefulness (5). The ingestion of caffeine in the evening can also affect the circadian rhythm by immediately reducing the level of melatonin (25).

Our results show that a higher proportion of girls consumed energy drinks than boys, which is not in line with previous studies that report that boys generally consume more than girls (17, 22, 24, 26). However, the gap is closing as consumption has been increasing more rapidly among girls (26). This trend is evident in an Icelandic survey, where more boys reported consuming energy drinks in 2016 and 2018, whereas by 2020, the trend had reversed, with more girls reporting consumption (20). Although our data show that fewer boys in total report drinking energy drinks (31 boys vs. 67 girls) we see higher percentage of boys exceeding the EFSA limits (2) of 1.4 and 3 mg caffeine/kg/day compared with girls. Similarly, we see that the boy’s total caffeine intake is 2.07 mg/kg/day but 1.77 mg/kg/day for girls. Alarmingly, more than 5% of boys consuming energy drinks exceeded the 5.7 mg/kg/day caffeine intake from all sources, which the EFSA panel has identified as the safety limit for adults, adolescents and children (2).

### Sleep and energy drink consumption

Overall, the participants generally did not follow sleep recommendations on weekdays for their age, as the NSF recommends 8–10 h of sleep for adolescents, and considers <7 h to be insufficient (9). Our participants’ total sleep duration on school days was 6.5 (1.1) hours per night, and 8.3 (1.1) hours per night on weekends. Our use of ≤6 h as a threshold reflects a more conservative criterion to identify adolescents with significant sleep restriction, which is supported by prior studies linking ≤6 h to negative outcomes (7, 12). This short sleep duration is consistent with earlier findings showing that the sleep patterns of Icelandic adolescents typically involve short durations, late bedtimes, and high night-to-night variability (27).

In general, sleep duration was shorter among participants who reported consuming energy drinks, and a greater proportion of this group reported sleeping less than 6 h per night on school days. However, it is interesting that the percentage of participants with a total sleep duration of 6 h or less was similar between those who consumed energy drinks but not after 3 pm and those who reported never consuming energy drinks. Overall, these results are in line with previous studies showing that drinking energy drinks can have an adverse effect on sleep (18, 19). However, although energy drink consumption was associated with shorter sleep duration, this association appeared weaker when consumption was limited to earlier in the day. It should also be observed that subgroup sizes were small in some of the higher caffeine intake groups, particularly those consuming ≥3 mg/kg/day. This limits statistical power and increases the likelihood of unstable estimates, so these findings should be interpreted cautiously and considered exploratory.

### Food and alcohol consumption

Energy drink consumption in previous studies has been related to several adverse outcomes and risk behaviors in adolescents (3), which is in line with the results from this study showing higher frequency of alcoholic beverage consumption among energy drink consumers compared with those who did not report drinking energy drinks.

The results also indicate that the food choices of energy drink consumers are poorer than those who do not consume energy drinks, including a higher intake of soft drinks. It should be observed that diet quality was in general far from dietary guidelines (28), both among energy drink consumers and those who did not consume energy drinks, with less than half of participants reporting daily intake of fruits, vegetables, and dairy products. This is of concern as healthy nutrition during adolescence can have significant long-term benefits as dietary habits formed during youth can influence nutritional behaviors in adulthood (29).

### Strength and limitations of the study

This study has several strengths. Firstly, the food frequency questionnaire was adapted from previously validated instruments, increasing the reliability of the dietary data. Secondly, the study provides detailed information on the timing and quantity of energy drink consumption, a behavior that has not been previously characterized in Icelandic adolescents. However, several limitations must be acknowledged. Firstly, the sample was recruited using convenience sampling from three secondary schools in the capital area, with a participation rate of 44%, which may limit the generalizability of the findings. Secondly, because participation was voluntary, the data may be subject to volunteer bias, for example, students with strong health interests or sleep concerns may have been more likely to respond. Thirdly, all data were collected using self-report questionnaires, including sleep behaviors, dietary intake, and anthropometric measures (height and weight), which introduces the potential for reporting bias and misclassification. While the questionnaires were based on validated tools, the exclusive reliance on self-reported data remains a methodological limitation. Additionally, data collection occurred during the COVID-19 pandemic, which may have influenced participants’ daily routines and lifestyle behaviors, including sleep and dietary patterns. Finally, the cross-sectional design limits the ability to draw conclusions about causality or directionality in the observed associations.

## Conclusions

In conclusion our study found that energy drink consumption was common among Icelandic adolescents, particularly among girls. Those who consumed energy drinks were more likely to report shorter sleep durations, especially when drinking after 3 PM. Additionally, energy drink consumers tended to have poorer dietary habits, with lower intake of nutritious foods and higher consumption of soft drinks, coffee, and alcohol. These findings suggest a potential link between energy drink consumption and both sleep and dietary behaviors in this population and highlights the need for further research to explore the long-term health impacts of energy drink consumption on sleep and diet. Future studies should examine the effectiveness of educational and policy interventions aimed at reducing energy drink intake and promoting healthier behaviors in adolescents.

## Supplementary Material



## References

[1] Nadeem IM, Shanmugaraj A, Sakha S, Horner NS, Ayeni OR, Khan M. Energy drinks and their adverse health effects: a systematic review and meta-analysis. Sports Health 2021; 13(3): 265–77. doi: 10.1177/1941738120949181 33211984 PMC8083152

[2] Nutrition and Allergies (NDA) EFSA Panel on Dietetic Products. Scientific opinion on the safety of caffeine. EFSA J 2015; 13(5): 4102. doi: 10.2903/j.efsa.2015.4102 PMC13151561 42109818

[3] Visram S, Cheetham M, Riby DM, Crossley SJ, Lake AA. Consumption of energy drinks by children and young people: a rapid review examining evidence of physical effects and consumer attitudes. BMJ Open 2016; 6(10): e010380. doi: 10.1136/bmjopen-2015-010380 PMC5073652 27855083

[4] Seifert SM, Schaechter JL, Hershorin ER, Lipshultz SE. Health effects of energy drinks on children, adolescents, and young adults. Pediatrics 2011; 127(3): 511–28. doi: 10.1542/peds.2009-3592 21321035 PMC3065144

[5] Aepli A, Kurth S, Tesler N, Jenni OG, Huber R. Caffeine consuming children and adolescents show altered sleep behavior and deep sleep. Brain Sci 2015; 5(4): 441–55. doi: 10.3390/brainsci5040441 26501326 PMC4701022

[6] Kristjansson AL, Sigfusdottir ID, Allegrante JP, James JE. Adolescent caffeine consumption, daytime sleepiness, and anger. J Caffeine Res 2011; 1(1): 75–82. doi: 10.1089/jcr.2011.00

[7] Owens J, Group ASW. Insufficient sleep in adolescents and young adults: an update on causes and consequences. Pediatrics 2014; 134(3): e921–32. doi: 10.1542/peds.2014-1696 25157012 PMC8194472

[8] Shochat T, Cohen-Zion M, Tzischinsky O. Functional consequences of inadequate sleep in adolescents: a systematic review. Sleep Med Rev 2014; 18(1): 75–87. doi: 10.1016/j.smrv.2013.03.005 23806891

[9] Hirshkowitz M, Whiton K, Albert SM, Alessi C, Bruni O, DonCarlos L, et al. National sleep foundation’s updated sleep duration recommendations: final report. Sleep Health 2015; 1(4): 233–43. doi: 10.1016/j.sleh.2015.10.004 29073398

[10] Garaulet M, Ortega FB, Ruiz JR, Rey-Lopez JP, Beghin L, Manios Y, et al. Short sleep duration is associated with increased obesity markers in European adolescents: effect of physical activity and dietary habits. The HELENA study. Int J Obes (Lond) 2011; 35(10): 1308–17. doi: 10.1038/ijo.2011.149 21792170

[11] Sivertsen B, Harvey AG, Lundervold AJ, Hysing M. Sleep problems and depression in adolescence: results from a large population-based study of Norwegian adolescents aged 16–18 years. Eur Child Adolesc Psychiatry 2014; 23(8): 681–9. doi: 10.1007/s00787-013-0502-y 24292341

[12] Lo JC, Ong JL, Leong RLF, Gooley JJ, Chee MWL. Cognitive performance, sleepiness, and mood in partially sleep deprived adolescents: the need for sleep study. Sleep 2016; 39(3): 687–98. doi: 10.5665/sleep.5552 26612392 PMC4763363

[13] Stefansdottir R, Rognvaldsdottir V, Chen KY, Johannsson E, Brychta RJ. Sleep timing and consistency are associated with the standardised test performance of Icelandic adolescents. J Sleep Res 2022; 31(1): e13422. doi: 10.1111/jsr.13422 34128282

[14] Chaput J-P, Tremblay A. Insufficient sleep as a contributor to weight gain: an update. Curr Obes Rep 2012; 1(4): 245–56. doi: 10.1007/s13679-012-0026-7

[15] Dewald JF, Oort FJ, Meijer AM. The effects of sleep extension on sleep and cognitive performance in adolescents with chronic sleep reduction: an experimental study. Sleep Med 2013; 14(6): 510–17. doi: 10.1016/j.sleep.2013.01.012 23523432

[16] Nuss T, Morley B, Scully M, Wakefield M. Energy drink consumption among Australian adolescents associated with a cluster of unhealthy dietary behaviours and short sleep duration. Nutr J 2021; 20: 1–10. doi: 10.1186/s12937-021-00719-z 34225738 PMC8259213

[17] Gunnarsdóttir S, Guðmannsdóttir R, Þorgeirsdóttir H, Torfadóttir J, Steingrímsdóttir L, Tryggvadóttir E, et al. Hvað borða Íslendingar? Könnun á mataræði Íslendinga 2019–2021: Helstu niðurstöður og samanburður við könnun frá 2010–2011. Reykjavík: Icelandic Directorate of Health & University of Iceland; 2022.

[18] Gardiner C, Weakley J, Burke LM, Roach GD, Sargent C, Maniar N, et al. The effect of caffeine on subsequent sleep: a systematic review and meta-analysis. Sleep Med Rev 2023; 69: 101764. doi: 10.1016/j.smrv.2023.101764 36870101

[19] Halldorsson T, Kristjansson A, Thorisdottir I, Oddsdóttir C, Sveinbjörnsson J, Benediktsson R, et al. Caffeine exposure from beverages and its association with self-reported sleep duration and quality in a large sample of Icelandic adolescents. Food Chem Toxicol 2021; 157: 112549. doi: 10.1016/j.fct.2021.112549 34509583

[20] Rannsóknir og greining. Ungt fólk 2020: framhaldsskólar. Reykjavík: Rannsóknir og greining; 2020.

[21] Kaldenbach S, Leonhardt M, Lien L, Bjærtnes AA, Strand TA, Holten-Andersen MN. Sleep and energy drink consumption among Norwegian adolescents – a cross-sectional study. BMC Public Health 2022; 22(1): 534. doi: 10.1186/s12889-022-12972-w 35303832 PMC8932303

[22] Svensson Å, Warne M, Gillander Gådin K. Longitudinal associations between energy drink consumption, health, and norm-breaking behavior among Swedish adolescents. Front Public Health 2021; 9: 597613. doi: 10.3389/fpubh.2021.597613 34178908 PMC8226087

[23] Miller KE, Dermen KH, Lucke JF. Caffeinated energy drink use by US adolescents aged 13–17: a national profile. Psychol Addict Behav 2018; 32(6): 647. doi: 10.1037/adb0000389 30124307 PMC6136946

[24] Jakobsdottir G, Stefansdottir RS, Gestsdottir S, Stefansson V, Johannsson E, Rognvaldsdottir V, et al. Changes in health-related lifestyle choices of university students before and during the COVID-19 pandemic: associations between food choices, physical activity and health. PLoS One 2023; 18(6): e0286345. doi: 10.1371/journal.pone.0286345 37352179 PMC10289399

[25] Burke TM, Markwald RR, McHill AW, Chinoy ED, Snider JA, Bessman SC, et al. Effects of caffeine on the human circadian clock in vivo and in vitro. Sci Transl Med 2015; 7(305): 305ra146. doi: 10.1126/scitranslmed.aac51 PMC4657156 26378246

[26] Puupponen M, Tynjälä J, Tolvanen A, Välimaa R, Paakkari L. Energy drink consumption among Finnish adolescents: prevalence, associated background factors, individual resources, and family factors. Int J Public Health 2021; 66: 620268. doi: 10.3389/ijph.2021.620268 34744582 PMC8565280

[27] Stefánsdóttir R, Rögnvaldsdóttir V, Gestsdóttir S, Guðmundsdottir SL, Chen KY, Brychta RJ, et al. Changes in sleep and activity from age 15 to 17 in students with traditional and college-style school schedules. Sleep Health. 2020; 6(6): 749–57. doi: 10.1016/j.sleh.2020.04.009 32534820 PMC7726031

[28] Blomhoff R, Andersen R, Arnesen EK, Christensen JJ, Eneroth H, Erkkola M, et al. Nordic nutrition recommendations 2023: integrating environmental aspects. Copenhagen: Nordic Council of Ministers; 2023.

[29] Thana’ YA, Takruri HR, Tayyem RF. Dietary practices and nutrient intake among adolescents: a general review. Obes Med 2019; 16: 100145. doi: 10.1016/j.obmed.2019.100145

