# Supplementary material for: Energy drink consumption, sleep behavior, and food choices of Icelandic adolescents
Source: Food Nutr Res. 2026 Jan 29;70:10.29219/fnr.v70.12190. doi: 10.29219/fnr.v70.12190 (PMC12893045; doi:10.29219/fnr.v70.12190)
Supplement: Supplementary file 1 [file FNR-70-12190-s1.docx]

Supplementary Table 1. Estimated portion sizes and caffeine content in one portion used for our analysis. Number and % of users of each type of energy drink.

| **Product** | **ml** | **Caffeine in one portion** | **Used by n** | **Used by %** |
| --- | --- | --- | --- | --- |
| Amino energy | 330 | 100 | 19 | 11 |
| Amino pro | 330 | 180 | 1 | 1 |
| Burn | 500 | 160 | 6 | 4 |
| Collab | 330 | 105 | 75 | 44 |
| Cult | 330 | 105 | 2 | 1 |
| Euroshopper energy drink* | 375 | 120 | 16 | 9 |
| Gogo | 330 | 105 | 1 | 1 |
| Monster | 500 | 160 | 38 | 22 |
| Monteindew | 500 | 80 | 11 | 6 |
| Nocco 105 | 330 | 105 | 72 | 42 |
| Nocco 180 | 330 | 180 | 26 | 15 |
| Orka | 500 | 160 | 1 | 1 |
| Reign | 500 | 200 | 7 | 4 |
| Ripped | 330 | 105 | 11 | 6 |
| Koffínvatn | 330 | 105 | 4 | 2 |
| Redbull | 250 | 80 | 35 | 20 |
| Coffee | 225 | 100 | 97 | 57 |
| Dark coloured soft drinks | 330 | 37 | 48 | 28 |

* The market share of Euroshopper in 250 ml (80 mg caffeine/portion) and 500 ml (160 mg caffeine per portion) is equal, therefore we used the average caffeine content of 120. When estimating the caffeine content of dark-colored soft drinks and coffee, we refer to the amounts used by the Icelandic authorities in a recent risk assessment related to caffeine intake ^30^
